# Molecular Characteristics of Human Adenovirus Type 3 Circulating in Parts of China During 2014–2018

**DOI:** 10.3389/fmicb.2021.688661

**Published:** 2021-06-29

**Authors:** Yali Duan, Baoping Xu, Changchong Li, Yixiao Bao, Shuhua An, Yunlian Zhou, Aihuan Chen, Li Deng, Limin Ning, Yun Zhu, Wei Wang, Meng Zhang, Lili Xu, Xiangpeng Chen, Zhengde Xie

**Affiliations:** ^1^Beijing Key Laboratory of Pediatric Respiratory Infection Diseases, Key Laboratory of Major Diseases in Children, Ministry of Education, National Clinical Research Center for Respiratory Diseases, Research Unit of Critical Infection in Children, Chinese Academy of Medical Sciences, 2019RU016, Laboratory of Infection and Virology, Beijing Pediatric Research Institute, Beijing Children’s Hospital, Capital Medical University, National Center for Children’s Health, Beijing, China; ^2^The 2nd Affiliated Hospital and Yuying Children’s Hospital of Wenzhou Medical University, Wenzhou, China; ^3^Xin Hua Hospital Affiliated to Shanghai Jiao Tong University School of Medicine, Shanghai, China; ^4^Children’s Hospital of Hebei Province, Shijiazhuang, China; ^5^The Children’s Hospital, Zhejiang University School of Medicine, Hangzhou, China; ^6^The First Affiliated Hospital of Guangzhou Medical University, Guangzhou, China; ^7^Guangzhou Women and Children’s Medical Center, Guangzhou, China; ^8^Children’s Hospital of Changchun, Changchun, China

**Keywords:** human adenovirus type 3, molecular characteristics, mutation, genetic recombination, acute respiratory infections

## Abstract

Human adenoviruses (HAdVs) are important pathogens causing respiratory infections; 3.5–11% of childhood community-acquired pneumonia is associated with HAdV infection. Human adenovirus type 3 (HAdV-3), leading to severe morbidity and mortality, is one of the most prevalent genotype among adenoviruses responsible for acute respiratory infections (ARIs) in children in China. To identify the genetic variation of HAdV-3 in children with ARIs in China, a molecular epidemiological study was conducted. A total of 54 HAdV-3 isolated strains were obtained from children with ARIs in Beijing, Wenzhou, Shanghai, Shijiazhuang, Hangzhou, Guangzhou, and Changchun from 2014 to 2018. Thirty-two strains of which were selected for whole-genome sequencing, while the hexon, penton base, and fiber genes were sequenced for remaining strains. Bioinformatics analysis was performed on the obtained sequences. The phylogenetic analyses based on whole-genome sequences, major capsid protein genes (hexon, penton base, and fiber), and early genes (E1, E2, E3, and E4) showed that the HAdV-3 strains obtained in this study always clustered together with the reference strains from Chinese mainland, while the HAdV-3 prototype strain formed a cluster independently. Compared with the prototype strain, all strains possessed nine amino acid (AA) substitutions at neutralization antigenic epitopes of hexon. The homology models of the hexon protein of the HAdV-3 prototype and strain BJ20160214 showed that there was no evident structural change at the AA mutation sites. Two AA substitutions were found at the Arg-Gly-Asp (RGD) loop and hypervariable region 1 (HVR1) region of the penton base. A distinct AA insertion (20P) in the highly conserved PPPSY motif of the penton base that had never been reported before was observed. Recombination analysis indicated that partial regions of protein IIIa precursor, penton base, and protein VII precursor genes among all HAdV-3 strains in this study were from HAdV-7. This study showed that the genomes of the HAdV-3 strains in China were highly homologous. Some AA mutations were found at antigenic sites; however, the significance needs further study. Our data demonstrated the molecular characteristics of HAdV-3 circulating in China and was highly beneficial for further epidemiological exploration and the development of vaccines and drugs against HAdV-3.

## Introduction

Human adenoviruses (HAdVs) belonging to the *Mastadenovirus* genus of the Adenoviridae family (Knipe and Howley, 2013) are double-stranded non-enveloped DNA viruses, which have been grouped into seven species (HAdV-A to G), with 104 genotypes^1^. HAdVs play a significant role in pediatric respiratory tract infections, especially in severe pneumonia, accounting for 3.5–11% of childhood community-acquired pneumonias (CAP) (Jain et al., 2015; Liu et al., 2015; Wang et al., 2015). HAdV infection can be severe, or even fatal, both in immunocompetent (Zarraga et al., 1992) and immunocompromised patients (Yu et al., 2016). Different HAdVs have different tissue tropisms correlating with different clinical manifestations. HAdV genotypes associated with respiratory infection diseases are species B (HAdV-3, 7, 11, 14, 16, 21, 50, and 55), C (HAdV-1, 2, 5, and 6), and E (HAdV-4) (Knipe and Howley, 2013).

HAdV-3, first isolated from a patient with acute respiratory infection (ARI) in the winter of 1952–1953, is one of the most prevalent serotypes responsible for respiratory infection diseases in children and adults worldwide (Lynch and Kajon, 2016). Our previous study indicated that HAdV-3, the predominant type of HAdV in China, accounted for 44.4% CAP caused by HAdV in children (Duan et al., 2019). Outbreaks of respiratory infections related to HAdV-3 have been reported many times. In 2011, an outbreak of febrile respiratory disease and pharyngoconjunctival fever in Hangzhou, China, was caused by HAdV-3 (Xie et al., 2012); in 2005, an outbreak of respiratory infection and conjunctivitis implicated in HAdV-3 infection occurred in a pediatric long-term care facility in Illinois, United States (James et al., 2007). A number of studies have demonstrated that HAdV-3 may lead to severe pneumonias in immunocompetent children (Rebelo-de-Andrade et al., 2010) and adults (Barker et al., 2003). The positive rates of neutralizing antibody against HAdV-3 showed an age-dependent increase. The seroprevalence against HAdV-3 was low (12.07–33.96%) in 1–5 year-old children, which was high (64.29–81.25%) in healthy adults (Tian et al., 2016, 2020). Furthermore, no effective vaccines or drugs for HAdV-3 are available. Therefore, monitoring the prevalence of HAdV-3 in China and analyzing the genetic stability and amino acid (AA) variation of the HAdV-3 genome, especially in the hypervariable regions 1–7 (loops 1 and 2) of the hexon gene, are of great importance to develop vaccines and drugs against HAdV-3.

To date, there are only 36 HAdV-3 complete genome sequences available in GenBank. It is inadequate to reflect the molecular epidemiological characteristics of HAdV-3. In this study, 54 HAdV-3 isolated strains were obtained from children with ARI in parts of China from 2014 to 2018, including Beijing, Wenzhou, Shanghai, Shijiazhuang, Hangzhou, Guangzhou, and Changchun. Thirty-two strains of HAdV-3 were randomly selected for whole-genome sequencing. Bioinformatics analyses of HAdV-3 sequences were conducted. Our study is in favor of promoting the understanding of the epidemiology and evolution of HAdV-3, as well as provide a foundation for the development of effective vaccines and public health strategies.

## Materials and Methods

### Strains

On the basis of a network monitoring viral pathogens of respiratory tract infections among children, samples positive for HAdV-3 from 2014 to 2018 were collected from parts of China. The sentinel hospitals in our surveillance network are distributed in northern China (Beijing Children’s Hospital and Children’s Hospital of Hebei Province), eastern China (Yuying Children’s Hospital, Xin Hua Hospital, and The Chiliren’s Hospital-Zhejiang University School of Medical), southern China (The First Affiliated Hospital of Guangzhou Medical University and Guangzhou Women and Children’s Medical Center), and northeastern China (Children’s Hospital of Changchun). The samples were collected from these sentinel points monthly. The inclusion criteria were uniform according to the guideline (Subspecialty Group of Respiratory Diseases The Society of Pediatrics; Chinese Medical Association The Editorial Board Chinese Journal of Pediatrics, 2013). The samples inoculated onto HEp-2 cells for virus isolation. A total of 54 strains were obtained, of which 32 strains were randomly selected for whole-genome sequencing and analysis. The information on strains is shown in Table 1.

**TABLE 1:** Isolates of HAdV-3 in this study.

**Strain name**	**Date of isolation**	**Place of isolation**	**Sequences obtained**	**GenBank accession numbers**
BJ20160214	2016	Beijing	Complete genome	MW748641
BJ20160246	2016	Beijing	Hexon, penton base, fiber gene	MW748610/MW748632/MW748588
BJ20170260	2017	Beijing	Hexon, penton base, fiber gene	MW748611/MW748633/MW748589
BJ20170262	2017	Beijing	Hexon, penton base, fiber gene	MW748612/MW748634/MW748590
BJ20170268	2017	Beijing	Hexon, penton base, fiber gene	MW748613/MW748635/MW748591
BJ20170281	2017	Beijing	Complete genome	MW748642
BJ20170284	2017	Beijing	Complete genome	MW748643
BJ20170287	2017	Beijing	Complete genome	MW748644
BJ20170306	2017	Beijing	Hexon, penton base, fiber gene	MW748614/MW748636/MW748592
BJ20170320	2017	Beijing	Complete genome	MW748645
BJ20170365	2017	Beijing	Hexon, penton base, fiber gene	MW748615/MW748637/MW748593
BJ20170371	2017	Beijing	Hexon, penton base, fiber gene	MW748616/MW748638/MW748594
BJ20170379	2017	Beijing	Complete genome	MW748646
BJ20170380	2017	Beijing	Hexon, penton base, fiber gene	MW748617/MW748639/MW748595
BJ20170382	2017	Beijing	Hexon, penton base, fiber gene	MW748618/MW748640/MW748596
BJ20180075	2018	Beijing	Complete genome	MW748647
BJ20180080	2018	Beijing	Complete genome	MW748648
BJ20180274	2018	Beijing	Complete genome	MW748649
BJ20180444	2018	Beijing	Complete genome	MW748650
BJ20180567	2018	Beijing	Complete genome	MW748651
BJ20180581	2018	Beijing	Complete genome	MW748652
BJ20180612	2018	Beijing	Complete genome	MW748653
BJ20180641	2018	Beijing	Complete genome	MW748654
BJ20180681	2018	Beijing	Complete genome	MW748655
BJ20180705	2018	Beijing	Complete genome	MW748656
BJ20180708	2018	Beijing	Complete genome	MW748657
BJ20180718	2018	Beijing	Complete genome	MW748658
BJ20180730	2018	Beijing	Complete genome	MW748659
BJ20180734	2018	Beijing	Complete genome	MW748660
BJ20180775	2018	Beijing	Complete genome	MW748661
CC20150103	2015	Changchun	Complete genome	MW748662
GZ20150033	2015	Guangzhou	Hexon, penton base, fiber gene	MW748597/MW748619/MW748575
GZ20150036	2015	Guangzhou	Complete genome	MW748663
GZ20150038	2015	Guangzhou	Hexon, penton base, fiber gene	MW748598/MW748620/MW748576
GZ20150047	2015	Guangzhou	Hexon, penton base, fiber gene	MW748599/MW748621/MW748577
HB20140057	2014	Shijiazhuang	Complete genome	MW748664
HB20150116	2015	Shijiazhuang	Hexon, penton base, fiber gene	MW748608/MW748630/MW748586
HB20150126	2015	Shijiazhuang	Complete genome	MW748665
HB20150330	2015	Shijiazhuang	Hexon, penton base, fiber gene	MW748609/MW748631/MW748587
SH20160050	2016	Shanghai	Hexon, penton base, fiber gene	MW748600/MW748622/MW748578
SH20160051	2016	Shanghai	Complete genome	MW748666
SH20160054	2016	Shanghai	Hexon, penton base, fiber gene	MW748601/MW748623/MW748579
SH20160055	2016	Shanghai	Complete genome	MW748667
WZ20150066	2015	Wenzhou	Complete genome	MW748668
WZ20150071	2015	Wenzhou	Hexon, penton base, fiber gene	MW748602/MW748624/MW748580
WZ20150072	2015	Wenzhou	Hexon, penton base, fiber gene	MW748603/MW748625/MW748581
WZ20150074	2015	Wenzhou	Hexon, penton base, fiber gene	MW748604/MW748626/MW748582
WZ20150076	2015	Wenzhou	Hexon, penton base, fiber gene	MW748605/MW748627/MW748583
WZ20150082	2015	Wenzhou	Complete genome	MW748669
WZ20150088	2015	Wenzhou	Hexon, penton base, fiber gene	MW748606/MW748628/MW748584
WZ20150089	2015	Wenzhou	Hexon, penton base, fiber gene	MW748607/MW748629/MW748585
ZJ20150106	2015	Hangzhou	Complete genome	MW748670
ZJ20150111	2015	Hangzhou	Complete genome	MW748671
ZJ20160114	2016	Hangzhou	Complete genome	MW748672

### Extraction of Viral Nucleic Acid

The viral nucleic acid was directly extracted from isolates using a QIAamp MinElute Virus Spin Kit (QIAGEN, Hilden, Germany) in line with the manufacturer’s instructions.

### Sequencing of Hexon, Penton Base, and Fiber Gene

Sequences of hexon, penton base, and fiber genes were amplified by polymerase chain reaction (PCR) using HotStar Taq Plus Master Mix Kits (QIAGEN, Hilden, Germany). The primers used in this study have been described earlier (Chen et al., 2015). PCR products were sequenced using a Sanger sequencing method by SinoGenoMax Co. Ltd. The sequences were assembled and edited with DNASTAR v7.1 (DNASTAR Inc., Madison, WI, United States). Hexon, penton base, and fiber genes were acquired from 54 strains.

### Sequencing of Whole Genome

A total amount of 700 ng DNA per sample was used as input material for the DNA sample preparations. Sequencing libraries were generated using NEB Next^®^ Ultra DNA Library Prep Kit for Illumina^®^ (NEB, Ipswich, MA, United States) following the manufacturer’s recommendations, and index codes were added to attribute sequences to each sample. Briefly, the chip DNA was purified using AMPure XP system (Beckman Coulter, Brea, CA, United States). After adenylation of the 3’ ends of DNA fragments, the NEB Next Adaptor with hairpin loop structure was ligated to prepare for hybridization. Electrophoresis was then used to select DNA fragments specified in length. USER Enzyme (NEB, Ipswich, MA, United States) was used with size-selected, adaptor-ligated DNA at 37°C for 15 min followed by 5 min at 95°C before PCR. PCR was then performed with Phusion High-Fidelity DNA polymerase, Universal PCR primers, and Index (X) Primer. At last, PCR products were purified (AMPure XP system), and library quality was assessed on the Agilent Bioanalyzer 2100 system. The clustering of the index-coded samples was performed on a cBot Cluster Generation System using HiSeq 4000 PE Cluster Kit (Illumina) according to the manufacturer’s instructions. After cluster generation, the library preparations were sequenced on an Illumina HiSeq 4000 platform, and 150-bp paired-end reads were generated. The quality controlled sequence data were *de novo* assembled using Shovill v1.0.9^2^ and BWA v0.7.17-r1188^3^ with minimum average coverage of 22.

### Phylogenetic Analysis

Whole-genomic reference sequences of HAdV-3 were downloaded from the GenBank database. Reference sequences typed as HAdV-3 only based on the hexon gene without the penton base or fiber genes were excluded. A total of 36 whole-genomic reference sequences obtained from GenBank were enrolled for phylogenetic analysis. Reference sequences of the hexon, penton, and fiber genes of HAdV-3 were also selected from the GenBank database for phylogenetic analysis. The reference sequences are listed in Table 2.

**TABLE 2:** Reference sequences used in this study.

**Accession Number**	**Year**	**Location**	**Sequences**
AY599834	1953	United States	Complete genome
AY599836	1997	United States	Complete genome
JX423380	2004	United States	Complete genome
JX423381	2003	United States	Complete genome
JX423382	2008	United States	Complete genome
KF268120	2007	United States	Complete genome
KF268123	2007	United States	Complete genome
KF268128	1988	United States	Complete genome
KF268131	2007	United States	Complete genome
KF268133	2007	United States	Complete genome
KF268202	Unknown	United States	Complete genome
KX384958	2002	United States	Complete genome
KY320276	Unknown	Korea	Complete genome
DQ099432	2005	Chinese mainland	Complete genome
DQ105654	2004	Chinese mainland	Complete genome
MK813914	2004	Chinese mainland	Complete genome
MK813915	2009	Chinese mainland	Complete genome
MK836308	2009	Chinese mainland	Complete genome
MK836310	2009	Chinese mainland	Complete genome
MK836311	2009	Chinese mainland	Complete genome
MK847517	2009	Chinese mainland	Complete genome
MK883603	2011	Chinese mainland	Complete genome
MK883604	2011	Chinese mainland	Complete genome
MK883608	2012	Chinese mainland	Complete genome
MW013769	2020	Chinese mainland	Complete genome
MW013770	2020	Chinese mainland	Complete genome
MW013771	2020	Chinese mainland	Complete genome
MW013772	2020	Chinese mainland	Complete genome
MW013773	2020	Chinese mainland	Complete genome
MW013774	2020	Chinese mainland	Complete genome
MW013775	2020	Chinese mainland	Complete genome
MW013776	2020	Chinese mainland	Complete genome
MW013777	2020	Chinese mainland	Complete genome
MW013778	2020	Chinese mainland	Complete genome
MW013779	2020	Chinese mainland	Complete genome
MW013780	2020	Chinese mainland	Complete genome
EF486498	Unknown	Taiwan, China	Hexon gene
EF486496	Unknown	Taiwan, China	Hexon gene
AF542127	Unknown	Korea	Hexon gene
AF542110	Unknown	Korea	Hexon gene
AF542104	Unknown	Korea	Hexon gene
AB900154	2004	Japan	Hexon gene
AB900151	2003	Japan	Hexon gene
AB900148	1988	Japan	Hexon gene
KM458630	2013	Chinese mainland	Hexon gene
KM458624	2012	Chinese mainland	Hexon gene
KM458623	2011	Chinese mainland	Hexon gene
KC456102	2011	Taiwan, China	Hexon gene
KC456097	2011	Taiwan, China	Hexon gene
KC456084	2011	Taiwan, China	Hexon gene
JQ764730	2011	Chinese mainland	Hexon gene
AB900153	2004	Japan	Penton base gene
AB900150	2003	Japan	Penton base gene
AB900147	1988	Japan	Penton base gene
KP270914	2013	Chinese mainland	Penton base gene
KP270908	2012	Chinese mainland	Penton base gene
KP270907	2011	Chinese mainland	Penton base gene
AB900155	2004	Japan	Fiber gene
AB900152	2003	Japan	Fiber gene
AB900149	1988	Japan	Fiber gene
AY224393	unknown	Korea	Fiber gene
AY224399	unknown	Korea	Fiber gene
AY224417	unknown	Korea	Fiber gene
KP270922	2013	Chinese mainland	Fiber gene
KP270917	2012	Chinese mainland	Fiber gene
KP270916	2011	Chinese mainland	Fiber gene
KC456119	2011	Taiwan, China	Fiber gene
KC456116	2011	Taiwan, China	Fiber gene
KC456112	2011	Taiwan, China	Fiber gene

MAFFT^4^ software was used to conduct multiple alignment of the hexon, penton base, fiber genes, and whole-genomic sequences. MEGA v6.0 (Sudhir Kumar, Arizona State University, Tempe, AZ, United States) software was used to generate phylogenetic trees with the neighbor-joining method and the Kimura two-parameter model. The robustness of the phylogenetic trees was assessed by the bootstrap method with 1,000 replicates.

### Analysis of Genetic Variation

The genetic variations of the hexon, penton base, and fiber genes were determined by BioEdit v7.2.0^5^ software.

The homology models of the hexon protein of the HAdV-3 prototype and strain BJ20160214 were built by submitting the AA sequences to SWISS-MODEL server^6^. The positions of mutated AA residues were labeled by PyMOL v2.4 in the HAdV-3 hexon structures.

### Recombination Analysis

SimPlot v3.5.1 software was used to complete similarity plots and recombination detection (bootscan approach) of the whole-genomic sequences of the HAdV-3. The default settings for window size, step size, distance model, and tree model were 1,000, 200, “Kimura,” and “Neighbor-Joining,” respectively.

## Results

### Phylogenetic Analysis of the Hexon, Penton Base, and Fiber Genes

The nucleotide and AA similarities of 54 strains obtained in this study were 99.8–100% and 99.6–100% for hexon, 99.8–100% and 99.7–100% for penton base, 99.5–100% and 99.6–100% for fiber.

**FIGURE 1:**
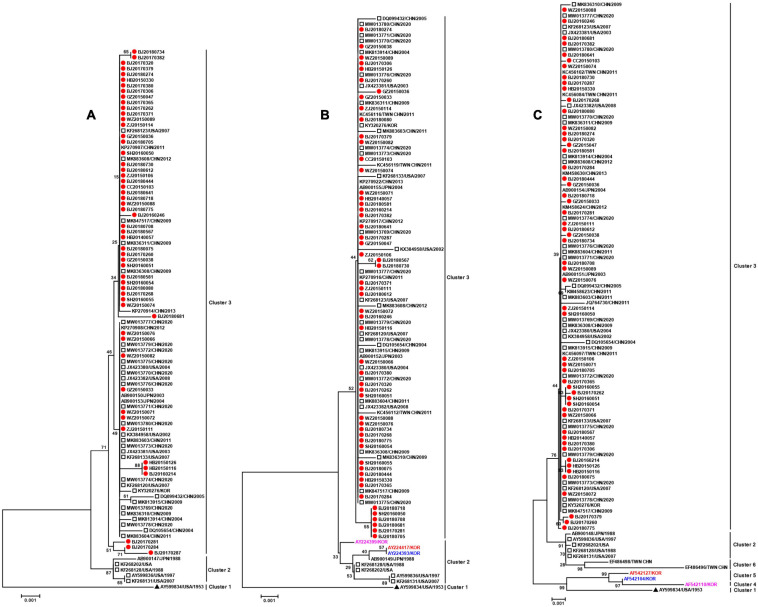
Phylogenetic analysis of the major capsid gene **(A)** penton base, **(B)** fiber, and **(C)** hexon. The phylogenetic trees were generated using the neighbor-joining method based on the Kimura two-parameter model with 1,000 replicates. The red dot indicates the strains obtained in this study. The black triangle indicates HAdV-3 prototype strain GB (accession number is AY599834). The empty square indicates the whole-genome reference sequences. AB900147, AB900148, and AB900149 are from the same strain.

Fifty-four penton base gene sequences obtained in this study and 42 reference sequences worldwide available from GenBank were analyzed by phylogenetic analysis. The phylogenetic tree of the penton base gene sequences formed three clusters. The prototype strain GB (AY599834) formed a separate cluster 1. Cluster 2 was constituted by four sequences from the United States and one sequence from Japan. All the strains circulating in China from 2004 to 2020 including 54 isolates obtained in this study as well as parts of strains circulating in the United States, Japan, and South Korea constituted cluster 3 (Figure 1).

The phylogenetic tree generated from 54 fiber gene sequences obtained in this study and 48 reference sequences worldwide available from GenBank formed three clusters. Cluster 1 and cluster 2 were consistent with the cluster 1 and cluster 2 in the phylogenetic tree formed by penton base. Similarly, the 54 isolates obtained in this study were in cluster 3 with all strains from China and part of strains from other countries (Figure 1).

A total of 105 hexon gene sequences were analyzed, including 54 sequences obtained in this study and 51 reference sequences worldwide downloaded from GenBank. As shown in the phylogenetic tree, 105 hexon gene sequences could be stratified into six clusters. Corresponding to the phylogenetic trees of penton base and fiber genes, the prototype strain GB (AY599834) was located in a distinct cluster 1; four strains from the United States and one strain from Japan comprised cluster 2; and all strains from mainland China were in cluster 3 including strains circulating in other countries or regions. Interestingly, three strains (AF542104, AF542110, and AF542127) from South Korea formed cluster 4 and cluster 5 in the hexon phylogenetic tree, respectively, however, these three strains all belonged to the cluster 2 in the phylogenetic tree of fiber gene, together with the epidemic strains from the United States and Japan. Cluster 6 was composed of two strains circulating in Taiwan, China.

### AA Variation Analysis

**FIGURE 2:**
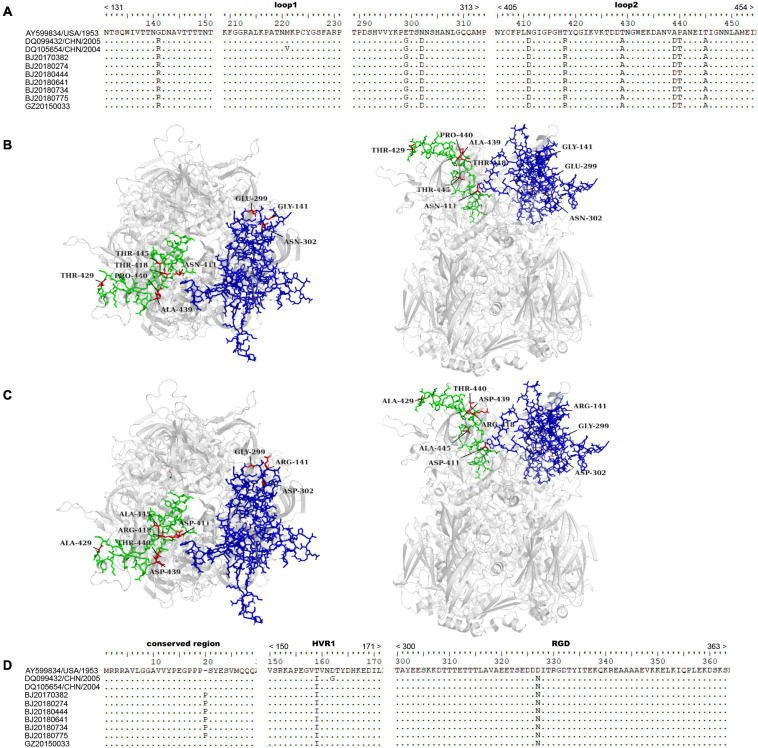
AA variation analysis in the loop 1 and loop 2 of hexon, RGD, HVR1, and conserved region of penton base. **(A)** AA variation in the loop 1 and loop 2 of hexon. **(B)** Structural model of HAdV-3 prototype hexon and **(C)** strain BJ20160214 hexon. Loop 1 and loop 2 are shown in blue and green, respectively. The mutation sites are shown in red (left, top view; right, side view). **(D)** AA variation in RGD, HVR1, and conserved region of penton base. In the HAdV-3 prototype strain GB, the region of loop 1 was 131–313 AA location of hexon, and that of loop 2 was 405–454 AA location of hexon (Pring-Akerblom et al., 1995); the region of RGD was 300–363 AA location of penton base, and that of HVR1 was 150–171 AA location of penton base (Madisch et al., 2007). The 54 hexon sequences we obtained were highly consistent and had same AA variations. Therefore, seven strains were selected as the representatives for AA variation analysis, and the strain BJ20160214 was selected for homology modeling.

After HAdV infection, the host produces neutralizing antibodies primarily targeting the neutralization epitopes on the surface of hexon. Antigenic domains and type-specific determinants have been mapped to loops 1 and 2 of the hexon and classified into seven hypervariable regions (HVRs): HVR1-6 (loop 1) and HVR7 (loop 2) (Sumida et al., 2005; Roberts et al., 2006). AA variations in the loop 1 and loop 2 were analyzed. Compared with the prototype strain GB, the 54 strains obtained in this study had three AA substitutions (G141R, E299G, and N302D) in the loop 1 region, and six AA substitutions (N411D, T418R, T429A, A439D, P440T, and T445A) in the loop 2 region. All reference sequences from China had same mutations in loop 1 and loop 2, except the reference sequence Guangzhou02 with an AA substitution (M221V) in loop 1. The homology models of the HAdV-3 prototype hexon and strain BJ20160214 hexon were built based on the AdC68 hexon crystal structure (PDB_ID 2OBE). The mutation sites were marked on the homology models (Figure 2). We aligned the two hexon model structurals and found that there was no distinct difference at the AA mutation sites of loop 1 and loop 2.

Compared with the prototype strain GB, an AA substitution (D327N) was observed at the Arg-Gly-Asp (RGD) loop in all strains circulating in China, and an AA substitution (T159I) was found at hypervariable region 1 (HVR1) in 54 isolates obtained in this study. However, another AA substitution (D162G) occurred in the reference sequence Guangzhou02, besides T159I. In addition, A distinct AA insertion (20P) was found in six isolates obtained in this study at the highly conserved PPPSY motif of the penton, which was not found in reference sequences (Figure 2).

### Phylogenetic Analysis of the Complete Genome

**FIGURE 3:**
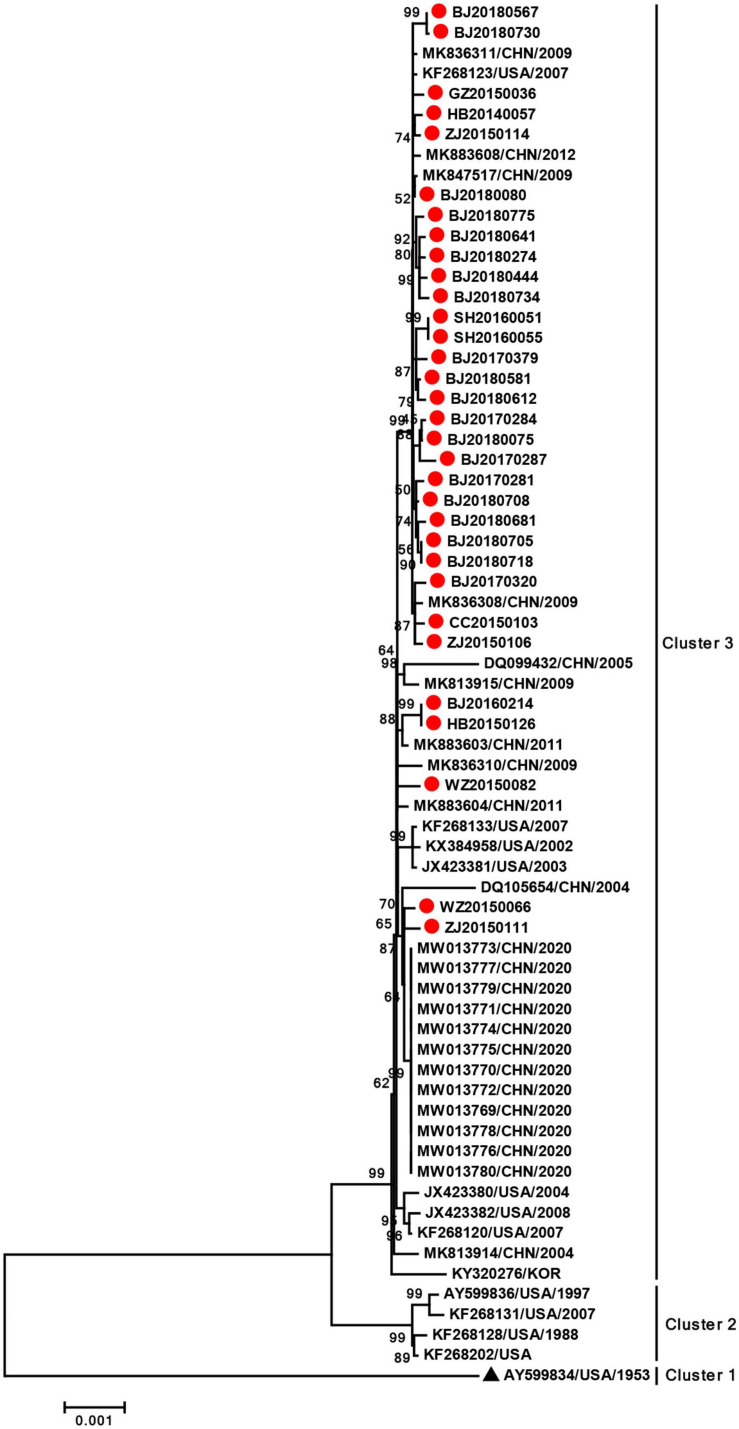
Phylogenetic analysis of complete genome of HAdV-3. The phylogenetic tree was generated using the neighbor-joining method based on the Kimura two-parameter model with 1,000 replicates. The red dot indicates the strains obtained in this study. The black triangle indicates HAdV-3 prototype strain (accession number is AY599834).

Since the analysis of the major capsid protein genes showed that strains we obtained were highly consistent, we randomly selected 32 strains for whole-genome sequencing and analysis. The nucleotide sequence identities were 99.3–100% among 32 HAdV-3 isolates. The phylogenetic dendrogram based on the whole-genome sequences formed three clusters. In accord with the phylogenetic tree of the major capsid, cluster 1 was formed by prototype strain GB. Cluster 2 consisted of four strains circulating in the United States from 1988 to 2007. The 32 isolates obtained in this study and reference strains circulating in China, United States, and Korea from 2002 to 2020 were located in cluster 3. Further, the 32 whole-genome sequences obtained in this study formed a small evolutionary branch supported by a significant bootstrap value in cluster 3, with a reference sequence collected from the United States, 2007 (Figure 3). These results indicated that strains obtained in this study had high percent identity and high homology.

### Phylogenetic Analysis of Early Genes

**FIGURE 4:**
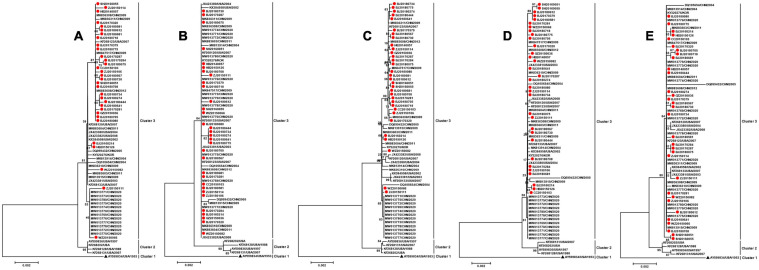
Phylogenetic analysis of the **(A)** E1, **(B)** E2A, **(C)** E2B, **(D)** E3, and **(E)** E4 genes. The phylogenetic tree was generated using the neighbor-joining method based on the Kimura two-parameter model with 1,000 replicates. The red dots indicate the strains obtained in this study. The black triangle indicates HAdV-3 prototype strain (accession number is AY599834).

The early genes of HAdV include E1, E2, E3, and E4 genes. Multiple recombination events of early gene regions occurred in species HAdV-C (Dhingra et al., 2019). The early genes play a crucial role in disturbing host immune defense mechanism as well as the transcription and replication of HAdV (Tauber and Dobner, 2001; Zeng and Carlin, 2019). In this study, the early genes were captured from the whole-genome sequences of 32 isolates for phylogenetic analysis. The phylogenetic trees based on the E1, E2A, E2B, E3, and E4 sequences formed three clusters, which were consistent with the tree based on whole-genome sequences. Similarly, clusters 1 and 2 were formed by the prototype strain GB and four strains circulating in the United States from 1988 to 2007, respectively. The 32 isolates obtained in this study were in cluster 3 together with the rest of the reference strains circulating in China, United States, and Korea from 2002 to 2020 (Figure 4).

### Recombination Analysis

**FIGURE 5:**
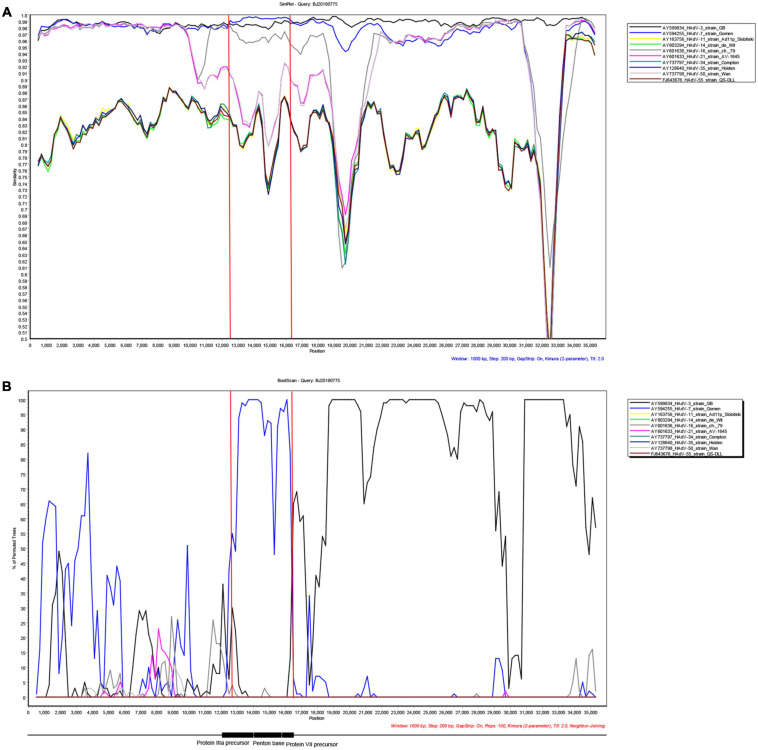
Genome recombination analysis.**(A)** SimPlot and **(B)** bootscan analysis of the whole genomes of strain BJ20180775 compared with other species B adenoviruses. Recombination analysis was performed by using SimPlot with the following inputs: window size [1,000 nucleotides (nt)], step size (200 nt), distance model (Kimura), and tree model (neighbor-joining). The GenBank accession numbers of prototype strains of each HAdV are as follows: HAdV-3, AY599834; HAdV-7, AY594255; HAdV-11, AY163756; HAdV-14, AY803294; HAdV-16, AY601636; HAdV-21, AY601633; HAdV-34, AY737797; HAdV-35, AY128640; HAdV-50, AY737798; and HAdV-55, FJ643676. The genome sequences of the 32 strains obtained in this study have high identity, and the results of the recombination analysis were consistent. Therefore, BJ20180775 was selected as the representative to display the results of the recombination analysis. Protein IIIa precursor, 12,051–13,817 nt gene location of prototype strain GB, without gaps. Penton base, 13,905–15,539 nt gene location of prototype strain GB, without gaps. Protein VII precursor, 15,553–16,131 nt gene location of prototype strain GB, without gaps.

Recombination plays a significant role in the evolution of HAdV. The recombination analysis of the 32 HAdV-3 whole-genome sequences demonstrated that a gene fragment coding partial protein IIIa precursor, penton base, and protein VII precursor were recombined from HAdV-7 (Figure 5). To further confirm the reorganization event, phylogenetic analysis of the recombinant region was performed, which showed that the recombinant region clustered with HAdV-7 (Supplementary Figure 1). The strain KF268128 isolated in 1988, the earliest strain among the reference sequences except the prototype strain GB, was analyzed by SimPlot. A similar recombination event was observed, indicating that the recombination event has taken place as early as 1988 (Supplementary Figure 2).

## Discussion

In the present study, phylogenetic analyses were performed for the 54 HAdV-3 isolates collected from seven cities in China between 2014 and 2018 for the hexon, penton base, and fiber sequences, and 32 of which were analyzed for the whole-genome sequences. Except hexon gene, which formed six clusters, the other genes all formed three clusters in the phylogenetic tree. Notably, all strains obtained from mainland China were together in the same cluster in the phylogenetic trees based on all genes, while the prototype strain GB was in a separate cluster. In addition, the nucleotide and AA identities of strains obtained in this study could reach a high degree ranging from 99.3 to 100%. Therefore, these results indicated that the HAdV-3 strains currently circulating in China had high identity and homology.

The hexon gene sequences were stratified into six branches, which may be correlated with heterogeneous HVRs of hexon. There are seven HVRs (HVR1–7) in the hexon protein, and their AA sequences are quite dissimilar in different types of HAdV but relatively conservative among different strains of one type HAdV. However, a research about variation in HVRs of HAdV-3 hexon reported that the HVRs of HAdV-3 strains were highly heterogeneous and could be categorized into 25 hexon variants, which was higher than the number of hexon variants of other types of HAdV (Haque et al., 2018). The heterogeneity of HVRs of hexon increased the challenge of developing a vaccine and drug for HAdV-3. Meanwhile, it prompted us to attach importance in analyzing the genetic variation characteristics of the HAdV-3, especially the genes coding antigen epitopes (such as the loop 1 and loop 2 of hexon).

The phylogenetic trees based on penton base, fiber, early genes, and genome all formed three clusters, while six clusters were separated in the hexon phylogenetic tree. These differences on the branch structures of phylogenetic trees based on different genes of the same strains prompted that these three strains, which formed cluster 4 and cluster 5 in the hexon phylogenetic tree may be derived from genetic recombination. Nevertheless, since there were no penton base and complete genome sequences of these three strains, we could not acquire further molecular biological information. Besides, two strains from Taiwan, China, formed the cluster 6 in the hexon phylogenetic tree. No further phylogenetic or recombination information could be obtained for the lack of penton base gene or genome. This suggests that we should strengthen the sequencing of the hexon, penton base, fiber, and the whole-genome sequences of HAdV in the future to obtain more epidemiological or genetic information on HAdV.

The RGD motif and HVR1, on the surface of the penton base, are type specific and hypervariable (Madisch et al., 2007). The RGD loop binds to the α_v_β_3_ or α_v_β_5_ integrins to facilitate the endocytosis process of the virus (Wickham et al., 1994). HVR1 may be a target of neutralizing antibodies. Recombination events around the HVR1 region has been reported (Madisch et al., 2007). Therefore, AA variation analysis at the RGD loop and HVR1 was conducted. Some AA mutations in the antigenic epitopes of hexon and HVR1 and RGD loop of penton base were found by comparing with the prototype strain GB. These mutations contained the substitutions of hydrophilic/hydrophobic AA (T429A, A439D, T445A, and T159I) and insertion of heterocyclic AA (20P). Similar AA substitutions in hexon have been observed in strains circulating in Japan, Korea, Germany, and Taiwan, China (Haque et al., 2018). The locations of the mutations in our study were not included in the epitopes that have been functionally confirmed in previous reports (Leen et al., 2004; Chakupurakal et al., 2013; Keib et al., 2017). However, we cannot completely rule out the influence of these mutations for antigenicity, which warrants further immunological investigations. It is worth noting that the insertion (20P) in the highly conserved PPPSY motif of penton base gene was discovered for the first time in this study. We analyzed and compared all penton base sequences available in GenBank, and no such insertion was observed. The PPPSY motif in this conserved region is an essential motif for dodecahedron penton particle structure (Zubieta et al., 2005). The influence of a heterocyclic AA (20P) insertion in PPPSY on the structural stability and other properties of penton base is currently unclear, and further studies are needed.

Recombination is a significant mechanism for the evolution of HAdV. Among the 104 HAdV genotypes we know, most HAdV new genotypes were produced by recombination (see text Footnote 1). Recombination analysis of 32 HAdV-3 strains in this study demonstrated that the partial region of protein IIIa precursor, penton base, and protein VII precursor of these strains were recombined from HAdV-7. Protein IIIa is a minor capsid protein of HAdV and responsible for stabilizing interactions between hexons and penton base (Liu et al., 2010; Reddy et al., 2010). Protein IIIa also plays a role in the packaging of viruses. It is involved in packaging virus DNA into the virus capsid (Ma and Hearing, 2011). Penton base is a major capsid protein, which is connected to the fiber. It contains the RGD loop region, which can interact with integrins to promote virus internalization (Wickham et al., 1994). The effect of recombination at this location on the structural stability, susceptibility, and virulence of the virus is still unclear, and further research is required.

In conclusion, this study comprehensively illustrated the molecular evolution characteristics of HAdV-3 circulating in China during 2014–2018. Our results revealed high homology of HAdV-3 circulating in China, and a few AA mutations were observed at antigenic epitopes of the hexon gene and RGD loop of the penton base gene. Whole-genome sequence analysis of currently circulating HAdV-3 strains in China indicated that partial regions of protein IIIa precursor, penton base, and protein VII precursor genes were recombined from HAdV-7, which might have occurred as early as 1988. Our research enriched the molecular epidemiology data of HAdV-3 in China and was highly conducive to further epidemiological exploration of HAdV-3-related severe clinical diseases and the development of vaccine and drugs against HAdV-3.

## Data Availability Statement

The datasets presented in this study can be found in online repositories. The names of the repository/repositories and accession number(s) can be found in the article/Supplementary Material.

## Author Contributions

YD, XC, and ZX conceived and designed the study, wrote the manuscript, and prepared the figures. YD, BX, CL, YB, SA, YZhou, AC, LD, LN, WW, and MZ performed the experiments. YD, XC, YZhu, and ZX analyzed the data. YD, XC, LX, and ZX checked and finalized the manuscript. ZX and XC provided resources. All authors read and approved the final manuscript.

## Conflict of Interest

The authors declare that the research was conducted in the absence of any commercial or financial relationships that could be construed as a potential conflict of interest.

## Footnotes

1 http://hadvwg.gmu.edu/

2 https://anaconda.org/bioconda/shovill

3 http://bio-bwa.sourceforge.net

4 https://www.ebi.ac.uk/Tools/msa/mafft/

5 https://bioedit.software.informer.com/download/

6 https://swissmodel.expasy.org/

## Supplementary Material

Supplementary Figure 1 Genome recombination analysis. **(A)** SimPlot and **(B)** bootscan analysis of the whole genomes of strain KF268128 compared with other species B adenoviruses. Recombination analysis was performed by using SimPlot with the following inputs: window size [1,000 nucleotides (nt)], step size (200 nt), distance model (Kimura), and tree model (neighbor-joining). The GenBank accession numbers of prototype strains of each HAdV are as follows: HAdV-3, AY599834; HAdV-7, AY594255; HAdV-11, AY163756; HAdV-14, AY803294; HAdV-16, AY601636; HAdV-21, AY601633; HAdV-34, AY737797; HAdV-35, AY128640; HAdV-50, AY737798; and HAdV-55, FJ643676. The genome sequences of the 32 strains obtained in this study have high identity, and the results of the recombination analysis were consistent. Therefore, BJ20180775 was selected as the representative to display the results of recombination analysis. Protein IIIa precursor, 12,051–13,817 nt gene location of prototype strain GB, without gaps. Penton base, 13,905–15,539 nt gene location of prototype strain GB, without gaps. Protein VII precursor, 15,553–16,131 nt gene location of prototype strain GB, without gaps.

Supplementary Figure 2 Phylogenetic analysis of recombinant region. Recombinant region: 12,286–16,193 nt gene location of prototype strain GB, without gaps. The phylogenetic tree was generated using the neighbor-joining method based on the Kimura two-parameter model with 1,000 replicates. The red dot indicates strains obtained in this study.
