# Supplementary figures and images for: Molecular Characteristics of Human Adenovirus Type 3 Circulating in Parts of China During 2014–2018
Source: Front Microbiol. 2021 Jun 29;12:688661. doi: 10.3389/fmicb.2021.688661 (PMC8276179; doi:10.3389/fmicb.2021.688661)

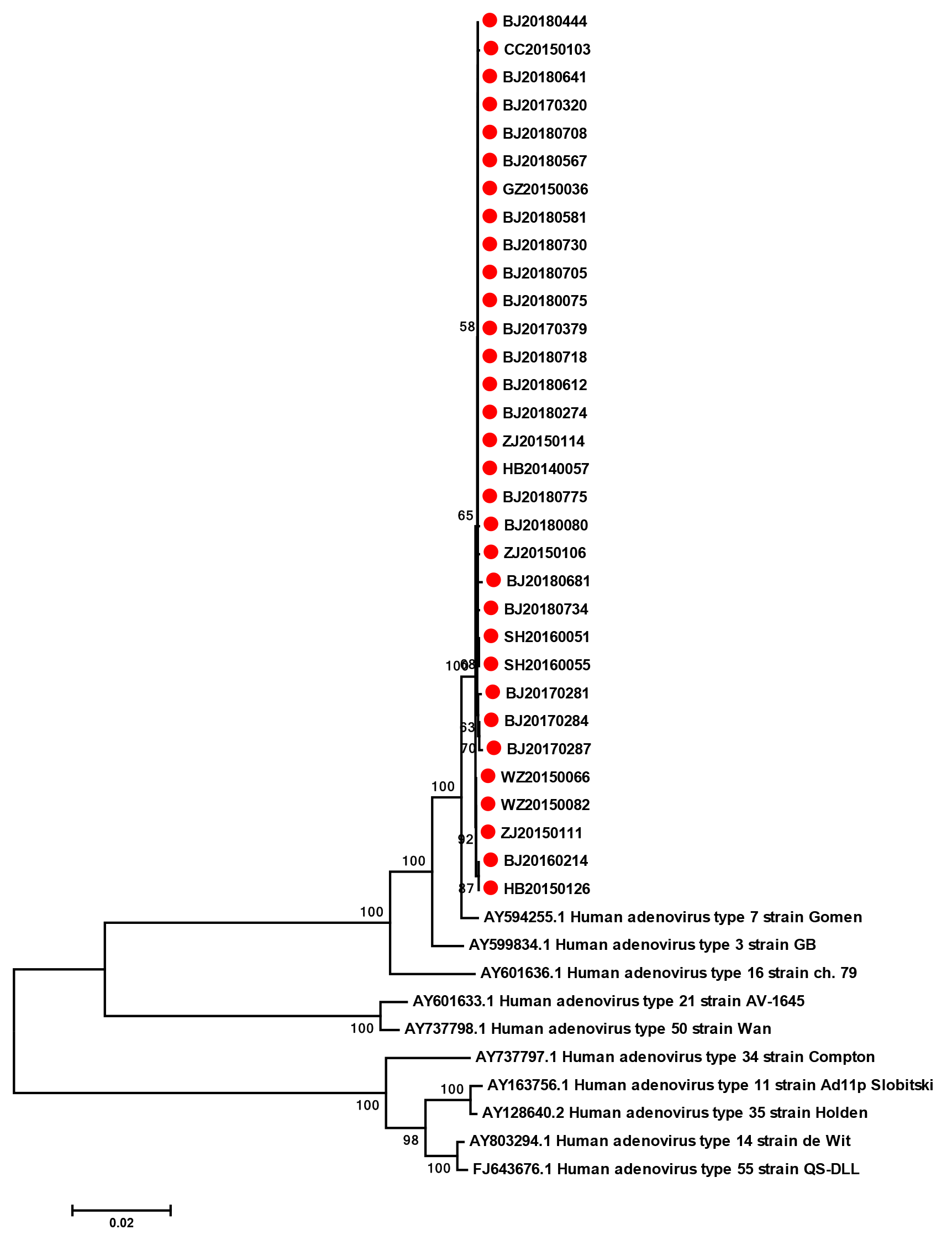

Supplement: Supplementary Figure 1 — Genome recombination analysis. (A) SimPlot and (B) bootscan analysis of the whole genomes of strain KF268128 compared with other species B adenoviruses. Recombination analysis was performed by using SimPlot with the following inputs: window size [1,000 nucleotides (nt)], step size (200 nt), distance model (Kimura), and tree model (neighbor-joining). The GenBank accession numbers of prototype strains of each HAdV are as follows: HAdV-3, AY599834; HAdV-7, AY594255; HAdV-11, AY163756; HAdV-14, AY803294; HAdV-16, AY601636; HAdV-21, AY601633; HAdV-34, AY737797; HAdV-35, AY128640; HAdV-50, AY737798; and HAdV-55, FJ643676. The genome sequences of the 32 strains obtained in this study have high identity, and the results of the recombination analysis were consistent. Therefore, BJ20180775 was selected as the representative to display the results of recombination analysis. Protein IIIa precursor, 12,051–13,817 nt gene location of prototype strain GB, without gaps. Penton base, 13,905–15,539 nt gene location of prototype strain GB, without gaps. Protein VII precursor, 15,553–16,131 nt gene location of prototype strain GB, without gaps. [file Image_1.TIF]

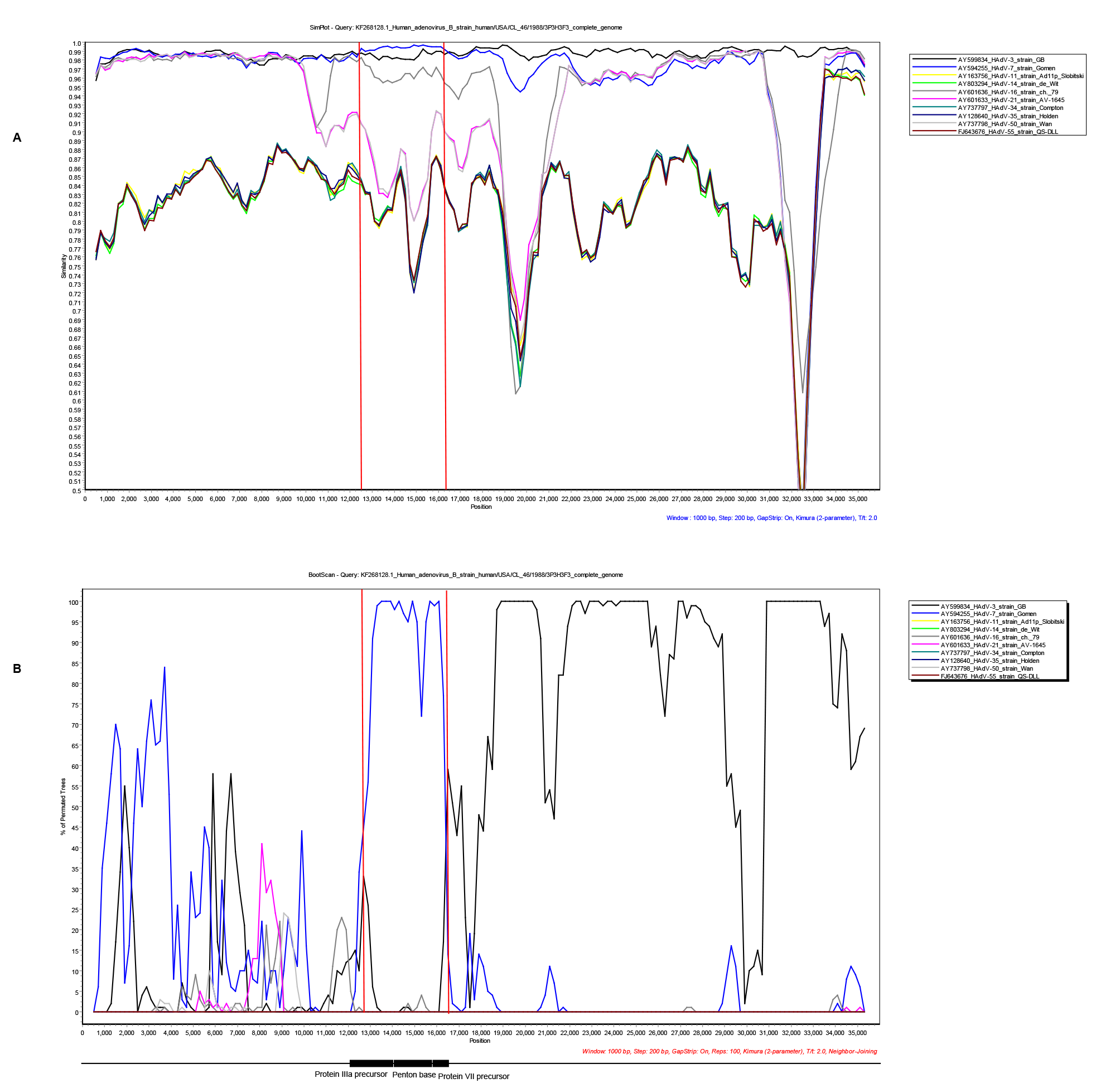

Supplement: Supplementary Figure 2 — Phylogenetic analysis of recombinant region. Recombinant region: 12,286–16,193 nt gene location of prototype strain GB, without gaps. The phylogenetic tree was generated using the neighbor-joining method based on the Kimura two-parameter model with 1,000 replicates. The red dot indicates strains obtained in this study. [file Image_2.TIF]
